# Influence of Moisturizers on Skin Microcirculation: An Assessment Study Using Laser Speckle Contrast Imaging

**DOI:** 10.3390/jpm13101507

**Published:** 2023-10-18

**Authors:** Ignace De Decker, Tanja Klotz, Peter Vu, Henk Hoeksema, Kimberly De Mey, Anse Beeckman, Bob Vermeulen, Marijn Speeckaert, Phillip Blondeel, Marcus Wagstaff, Stan Monstrey, Karel E. Y. Claes

**Affiliations:** 1 Burn Center, Ghent University Hospital, C. Heymanslaan 10, 9000 Ghent, Belgium; hendrik.hoeksema@uzgent.be (H.H.); kim.demey@uzgent.be (K.D.M.); phillip.blondeel@ugent.be (P.B.); stan.monstrey@ugent.be (S.M.); karel.claes@uzgent.be (K.E.Y.C.); 2 Department of Plastic Surgery, Ghent University Hospital, C. Heymanslaan 10, 9000 Ghent, Belgium; 3 Adelaide Medical School, The University of Adelaide, Adelaide, SA 5000, Australia; tanja.klotz@sa.gov.au (T.K.); marcus.wagstaff@sa.gov.au (M.W.); 4 Department of Occupational Therapy, Royal Adelaide Hospital, Port Rd., Adelaide, SA 5000, Australia; 5 SA Pharmacy, Royal Adelaide Hospital, Adelaide, SA 5000, Australia; peter.vu@sa.gov.au; 6 Faculty of Medicine and Health Sciences, Sint-Pietersnieuwsstraat 33, 9000 Ghent, Belgium; anse.beeckman@ugent.be; 7 Department of Plastic Surgery, Ziekenhuis Oost-Limburg, Synaps Park 1, 3600 Genk, Belgium; 8 Department of Nephrology, Ghent University Hospital, C. Heymanslaan 10, 9000 Ghent, Belgium; marijn.speeckaert@ugent.be; 9 Adult Burns Service and Department of Plastic Surgery, Royal Adelaide Hospital, Port Rd., Adelaide, SA 5000, Australia

**Keywords:** moisturizers, scar, fluid silicone gel, laser speckle contrast imaging, occlusion, hydration, flux, skin perfusion, hypertrophic scars, scar management

## Abstract

Non-invasive scar management typically involves pressure therapy, hydration with silicones or moisturizers, and UV protection. Moisture loss from scars can lead to hypertrophic scar formation. Pressure therapy reduces blood flow, fibroblast activity, and transforming growth factor beta 1 (TGF-β1) release. This study examined various moisturizers and liquid silicone gel’s impact on microcirculation. 40 volunteers participated in a study where superficial abrasions were created to induce trans epidermal water loss (TEWL). Five moisturizers (TEDRA^®^, TEDRA^®^ NT1, TEDRA^®^ NT3, Alhydran^®^, Lipikar^®^) and BAP Scar Care^®^ silicone gel were tested. TEWL, hydration, and blood flow were measured up to 4 h post-application. Results showed that silicone had the least impact on occlusion and hydration. Alhydran^®^ reduced blood flow the most, while Lipikar^®^ increased it the most. TEDRA^®^ NT1 had reduced flow compared to TEDRA^®^ and TEDRA^®^ NT3. All TEDRA^®^ products exhibited high hydration, and all but silicone showed good occlusion. Moisturizers influenced skin microcirculation, with some causing decrease, while others increased flow. However, the clinical impact on scarring remains unclear compared to the evident effects of hydration and occlusion. More research is necessary to study moisturizers alone and with pressure therapy on scars, along with potential adverse effects of increased microcirculation on scars.

## 1. Introduction

Moisturizers and silicone-containing lotions are commonly recommended by burn specialists for hypertrophic scars (HTS) as part of a scar management regimen whilst the scar is active. Furthermore, in certain cases, it is advised to continue life-long moisturization, even after the scars have fully matured, to maintain the skin, as natural moisturization and skin hydration might prove insufficient [1]. HTS exhibit distinct characteristics, including thickening, rigidity, an erythematous appearance, increased cellular patterns, a disorganized collagen fiber network and hypervascularity, which distinguishes them from normal skin [2]. In the active phase, hypertrophic scars exhibit increased trans epidermal water loss (TEWL) attributed to the thinner and less matured stratum corneum [3]. The elevated TEWL in active scars has been correlated with other invasive (i.e., histology and immunohistochemistry) and non-invasive measures of scar activity [4]. This increased TEWL stimulates stratum corneum keratinocytes to signal the fibroblasts, via cytokines, to synthesize and release collagen, resulting in hypertrophic scarring [5,6]. Contact media, such as silicone gels and sheets, are also part of this regimen. The assumed mechanism of action of contact media is the normalization of elevated TEWL, which is widely accepted [5]. Normalizing the TEWL can downregulate scar activity, resulting in improved cosmetic and functional outcomes, making it a fundamental aspect of scar treatment protocols [6,7].

Elevated TEWL is not only present in active scars but can also be present in other conditions, such as atopic dermatitis and psoriasis, where the integrity of the stratum corneum is compromised and can even be influenced by skin type, preventive measures or use of cosmetics [8,9]. Various skin types can manifest unique TEWL profiles, including dry, dehydrated and oily skin, or a combination thereof [8,9]. TEWL measurement is widely accepted in dermatological research as a means of assessing stratum corneum function and is utilized in studies investigating the effects of topical products, skin dysfunction, and disease [9,10]. In addition to TEWL, hydration or water content of the skin have been extensively reported in the dermatological literature [11]. Both TEWL and hydration play significant roles in influencing the biophysical properties of the stratum corneum. Hydrated keratinocytes have been shown to decrease type I collagen production from dermal fibroblasts through paracrine interactions [12,13]. Conversely, decreasing keratinocyte hydration results in the activation of dermal fibroblast cytokines, resulting in increased collagen production and ultimately contributing to hypertrophic scar formation [14].

The tape stripping model has been widely recognized as a research technique for replicating a compromised stratum corneum [15]. This model provides a replicable approach to elevate TEWL under in vivo conditions, enabling assessment of the effects of products applied to the skin. This technique has previously been used in studies to examine the effects of moisturizers [16,17,18]. The results of these studies demonstrated that the elevated TEWL from tape stripping could be reduced, approaching normalization, with the application of certain selected moisturizers. However, the more expensive fluid silicone gels used in these studies were found to be less effective in decreasing TEWL and increasing hydration based on the Tewameter and Corneometer, respectively, suggesting that they may have limited efficacy in clinical applications [16,18].

The favorable effects of moisturizers on TEWL have been documented in previous studies. De Paepe et al. (2015) investigated the effects of petrolatum and silicone-containing creams [17]. Both decreased TEWL, but only petrolatum was able to significantly hydrate the skin [17]. Loden et al. (1997) found that application of a moisturizer decreased TEWL on Sodium Lauryl Sulphate-damaged skin (SLS); a known irritant to increase TEWL [19]. Similar early research, conducted on hairless mice, showed that the application of moisturizers decreased elevated TEWL and improved skin hydration (conductance) [20].

In addition to exhibiting elevated TEWL, active scars are highly perfused, as clinically observed through their erythematous appearance. The application of pressure to reduce perfusion of the active scar to induce a relative ischemia, thereby impairing the growth of fibroblasts and inhibiting their potential transformation into myofibroblasts, is a well-recognized scar management technique [21,22]. Furthermore, the application of mechanical pressure has been demonstrated to decrease the expression of transforming growth-factor-β1 (TGF-β1), which is an essential fibroblast activity regulator [23,24,25]. Pressure garments are frequently considered amongst the first line of scar management techniques [21,26,27]. As a result, the degree of perfusion and erythema has been measured in research using subjective and objective methods. Objective measurements of erythema can include devices capable of quantifying redness, as well as lasers which detect blood flow, such as Laser Doppler Imaging (LDI) and Laser Speckle Contrast Imaging (LSCI). Stewart et al. (2005) compared LSCI with LDI when measuring hypertrophic scar perfusion and were able to show a positive correlation (r^2^ = 0.86) [28]. This study also noted that the LSCI yielded faster scan times and higher resolution [28]. Yang et al. (2021) demonstrated that blood perfusion as measured with LSCI is increased in hypertrophic and keloid scars compared to normal skin [29]. In addition, LSCI was sensitive enough to detect lower perfusion in the central region of keloid scars compared to the marginal region [29].

LSCI devices have been developed for medical and non-medical applications over the last three decades, including intra- and post-operative flap monitoring, burn depth assessment, cerebral blood flow and retinal perfusion [30,31]. LSCI involves the projection of coherent laser light onto the skin, which is then scattered to form a speckle pattern on the detector surface of a video camera. The movement of blood within the tissue causes the speckle pattern to move so that, the faster the blood flow, the faster the speckle moves, allowing for analysis of the variations to objectively measure perfusion [30].

In their 2016 study [32], Liu and colleagues employed Laser Speckle Contrast Imaging (LSCI) to assess perfusion in keloids as well as in the surrounding and non-adjacent skin. Their findings unveiled a notable twofold increase in perfusion within the scar tissue. Chen et al. (2021) [33] from the same institution replicated this study with the LSCI to also show an approximate doubling of the perfusion units of a keloid scar compared to normal skin.

Hypertrophic scars and other skin conditions have elevated TEWL and increased perfusion, as previously demonstrated. Clinicians commonly prescribe the application of products to hydrate the impaired skin and reduce TEWL. In the Belgian context, Alhydran^®^ (BAP Medical, Apeldoorn, The Netherlands) and Dermacress (Cressana, Zwalm, Belgium) are recommended moisturizers. Additionally, the Belgian Burn Foundation proposed TEDRA^®^ (OLys Pharma, Verlaine, Belgium), a water-based moisturizer, for patients with burn scars. The foundation prioritizes affordability, and it aims to ensure that patients have access to high-quality care without an excessive financial burden. Currently TEDRA^®^ is being recommended as an alternative moisturizing cream, alongside Alhydran^®^ and Dermacress. All the separate ingredients of the formulations used in this study and their respective function can be found in Appendix A.

To expand upon previous work [18], the primary objectives of this study were to explore the potential relationship between the application of selected moisturizers on tape-stripped skin and cutaneous blood flow, as well as to examine the interplay between this relationship and occlusion and hydration.

## 2. Materials and Methods

### 2.1. Ethics Committee

The ethical committee of Ghent University Hospital granted approval for this study on 15 June 2022 (Belgian registration number B6702022000247). Before enrolling in the study, both oral and written informed consent were obtained from every healthy volunteer.

### 2.2. Power Analysis

In order to evaluate the non-inferiority of TEDRA^®^ in comparison to Alhydran^®^, utilizing the confidence interval method for the mean difference in TEWL reduction (with a constructed 95% CI) from data gathered in a paired design, and referencing the prior study by De Decker et al. (2022) [16], a sample size of 36 patients receiving treatment with both Alhydran^®^ and TEDRA^®^ was selected. This sample size yields a statistical power of 90% under the assumption that there is no true difference (i.e., both products are equivalent), the standard deviation is 0.097, and the non-inferiority margin is 0.05. To account for possible dropouts, an additional 10% (4 volunteers) was included, resulting in a total of 40 participants.

### 2.3. Scar-Like Model

In this study a similar, standardized, reliable, and uniform scar-like model with increased TEWL was used, as in previous studies [17,18].

The main focus of this study was to assess the influence on skin microcirculation using LSCI, in addition to investigating the occlusive and hydrative properties of moisturizers, as examined in the studies by Hoeksema et al. (2013) [18] and De Decker et al. (2022) [16], which focused on the occlusive and hydrative properties of moisturizers.

#### 2.3.1. Participant Recruitment

Young, healthy adult volunteers aged 20–35 years with intact skin on their inner forearms were eligible for participation in this study. Inclusion of this age group ensured a comparable thickness of the skin. The exclusion criteria included tattoos in the region of interest, wound healing or active scarring in the region of interest, dermatological disorders, metabolic conditions affecting the skin, and medication altering the hydration state of the stratum corneum.

**Figure 1:**
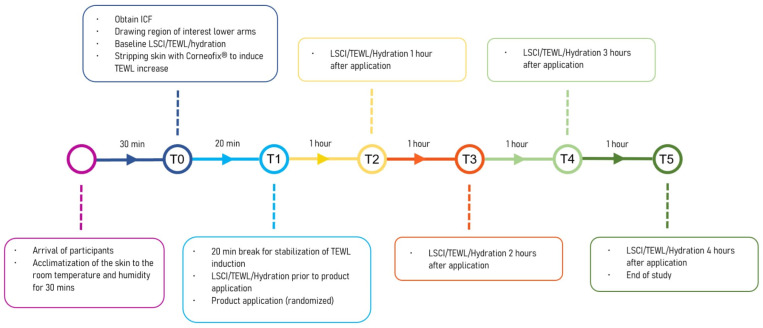
Flowchart of the study protocol. TEWL: Trans-epidermal Water Loss. LSCI: Laser Speckle Contrast Imaging.

Two weeks prior to the start of the study, the participants were asked not to use hydrating products and cease additional skincare routines. Participants were instructed to refrain from showering, using creams or lotions, and consuming coffee, soft drinks, or smoking on the night before and morning of the study, as well as during the experiment. Upon arrival, the volunteers were required to remove any clothing that covered their forearms. A 30 min acclimatization period was implemented for allowing study participants to adjust to the room temperature and humidity. The temperature and humidity of the room were continuously assessed using an ambient condition sensor, RHT 100 (Courage + Khazaka electronic GmbH, Köln, Germany). During the acclimatization period, 4 areas of 2 × 2 cm were drawn with a black marker (Staedtler permanent Lumocolor, Nürnberg, Germany) on the volar side of each of the forearms, starting 2 cm distal from the elbow and spaced 2 cm apart, ensuring no product mixing during application. Sites with moles or a prominent superficial venous network were avoided by translating the drawn areas slightly laterally. In male volunteers, excessive hair on designated areas was shaved with a hair clipper. The study protocol is illustrated in a chronological flowchart in Figure 1.

#### 2.3.2. Tape Stripping Procedure

After the acclimatization period, the initial set of measurements was taken at all 8 marked zones (T0), followed by the stripping process to induce superficial abrasions in 7 of the marked zones. This skin-stripping model was studied by Hoeksema et al. (2013) [18] and De Decker et al. (2022) [16]. A special adhesive tape, Corneofix CF 20 (Courage + Khazaka Electronic GmbH, Köln, Germany), measuring 2 by 2 cm, which collects corneocytes (flakes of dead cells), was used. The arms were kept in a maximal extended and supine position with closed fists to enable a stable and even surface for Corneofix application. The designated areas for the unstripped and stripped controls were the first and second most proximal areas respectively of either the left or right arm, and this according to the randomization process. In a previous study conducted by De Decker et al. (2022) [16], both stripped and unstripped control areas were examined on each arm. However, upon obtaining comparable data from the control sites on each arm, the decision was made in this study to focus on only one stripped and one unstripped control area. The remaining areas were preserved for stripping and subsequent product application, also according to the randomization process. To apply the Corneofix patch, a purpose-designed stamp was applied for three seconds for extra pressure and to ensure full and equal adhesion on the skin. Subsequently, the Corneofix was removed. This process was repeated 20 times for each stripping area. The adequacy of stripping could be assessed by observing the number of corneocytes per strip. Immediately after stripping, a demarcated slightly moist and erythematous surface was observed. A waiting time of 20 min was used to induce TEWL stabilization. A second set of measurements was taken at 20 min post-stripping (T1).

#### 2.3.3. Product Application and Measurements

The randomization of the products was determined using online software (Research Randomizer Version 4.0, Scott Plous, Lancaster, PA, USA). Ten days of experiments, each with four participating volunteers, were organized. The same randomization scheme was used for all the participants on the same day. After randomization, the clinician’s hands were washed, disinfected, and air-dried. A sufficient amount of product was then taken from the container to cover an area of 4 cm^2^. Using the index finger, the product was evenly spread in a smooth circular motion, without interfering with other designated areas. Any remaining product from the previous application was removed by wiping it away with a dry tissue. The hands were again disinfected and air-dried, and a different finger was used to apply the next product. Once all products were applied, the volunteers were instructed to remain still and avoid touching the test zones. The measurements of microcirculation, TEWL (transepidermal water loss), and hydration were taken in a sequential order, with hourly intervals up to four hours following the application of the product. These measurement time points were labelled as 1 h:T2, 2 h:T3, 3 h:T4, and 4 h:T5. Figure 1 illustrates the chronological sequence of product application and the corresponding measurement intervals.

At the end of the study period, the participants were provided with moisturizer samples. They were instructed to apply the products twice daily until ‘healing’ was achieved in all stripped areas.

### 2.4. Research Equipment

#### 2.4.1. Laser Speckle Contrast Imaging (LSCI)

The microcirculation in both arms was measured using Laser Speckle Contrast Imaging (LSCI; FLPI-2, Moor Instruments Ltd., Axminster, UK). The LSCI measurements are expressed in arbitrary perfusion units (P.U.) The FLPI-2 scanner was switched for a minimum of 30 min prior to the first scan to warm up the probe and avoid any possible deviations due to undercooling. To ensure the absence of possible bias from circulation stimulation by probing the skin, the LSCI scan was always performed before the other measurements with the Tewameter^®^ and Corneometer^®^.

Given the rather limited scannable surface with the LSCI scan, both arms were scanned separately at each time point. Labels for each study participant, indicating both the study number, arm (left vs. right) and timepoint (T0–T5), were made and placed in the ‘to scan’ area. Prior to scanning, the volunteers were asked to remain as still as possible to avoid perfusion deviation due to movement. Light scattering was avoided due to the absence of windows in the examination room. The quality of the scan was immediately assessed and, when artifacts or confounding factors that could affect the accuracy were present such as motion or external interference, a new scan was performed. For the majority of scans (>95%), a re-scan was not required. The software MoorFLPI-2 Clinical 1.0 (FLPI-2, Moor Instruments Ltd., Axminster, UK) was used for scan analysis.

For final analysis, each region of interest (ROI) was manually selected within a standardized squared designated area of 2 cm × 2 cm. When analyzing the ROIs for perfusion values, the marker points of the black highlighter were excluded, as the marker partially absorbed the emitted infrared signals. Average flux values and the standard deviations for each ROI at every timepoint (T0–T5) were extracted from the scans using MoorFLPI-2 Clinical V1.0 software.

After the LSCI scan, measurements were taken using a Tewameter TM 300^®^ (Courage + Khazaka Electronic GmbH, Köln, Germany) and Corneometer CM 825^®^ (Courage + Khazaka Electronic GmbH, Köln, Germany). LSCI was chosen over Laser doppler Imager (LDI) given the faster scan times and superior mobility compared to LDI.

#### 2.4.2. Tewameter TM 300^®^

The Tewameter^®^ TM 300 (Courage + Khazaka Electronic GmbH, Köln, Germany) was used to measure TEWL. The Tewameter is based on the diffusion principle in an open chamber, which is the only method to assess TEWL continuously without influencing the skin surface. Six seconds of measurement with a Tewameter^®^ was performed to stabilize the TEWL value and reduce variability. Six values were obtained and the mean values were recorded. The probes were cleaned using dry cloth between the switching locations. The probes were rinsed with an alcoholic swab between the volunteers. TEWL is expressed in g/h/m^2^. The CK Multi Probe Software (Courage + Khazaka Electronic GmbH, Köln, Germany) was used for data collection from the Tewameter.

#### 2.4.3. Corneometer CM 825^®^

Skin hydration was measured using a Corneometer CM 825^®^ (Courage + Khazaka Electronic GmbH, Köln, Germany). Six measurements with the Corneometer^®^ were taken at different locations within the designated study area. Each time, the mean values of the six measurements were recorded and used for subsequent data collection. The hydration values are expressed in arbitrary units (A.U.). Between switching locations, the probes were cleaned with a dry cloth. Additionally, the probes were rinsed with an alcohol swab between volunteers. The CK Multi Probe Software (Courage + Khazaka Electronic GmbH, Köln, Germany) was used for data collection from the Corneometer.

#### 2.4.4. Products

In this study, six different products were tested, including five moisturizers and one fluid silicone gel: Alhydran^®^ (BAP Medical, Apeldoorn, The Netherlands), TEDRA^®^ (Olys^®^ Pharma, Verlaine Belgium), TEDRA^®^ NT1 (Olys^®^ Pharma, Verlaine, Belgium), TEDRA^®^ NT3 (Olys^®^ Pharma, Verlaine, Belgium), (Lipikar^®^ Baume AP+M, La Roche-Posay, France) and BAP Scar Care fluid silicone gel^®^ (BAP Medical, Apeldoorn, The Netherlands).

In our burn center, Alhydran^®^ is considered the standard of care. It is an oil emulsified in water and is mainly composed of Aloe Vera Gel, which provides a moisturizing effect [34]. The other primary ingredients of Alhydran^®^ are Jojoba oil, cetearyl alcohol and caprylic triglyceride. Alhydran^®^ has an anti-itch effect, as demonstrated in a study conducted in the Netherlands [34]. The mechanism of action is a combination of the moisturizing effect of the Aloe Vera gel with an occlusive and emollient effect provided by the fatty ingredients, such as cetearyl alcohol and caprylic triglyceride, and both can be obtained naturally, are present in many skincare products and are often used in formulations for sensitive skin [17,34,35,36].

Lipikar^®^ Baume AP+M, which primarily consists of water, niacinamide, shea butter and aqua posae filiformis, is a hydrating moisturizer, which has an anti-pruritic and photo-protective properties mostly attributable to the incorporated niacinamide, which is an amide of vitamin B3 (niacin) [37,38]. Niacinamide has been demonstrated to be more effective than white petroleum in decreasing TEWL and increasing hydration [39]. Moreover, it helps to protect the lipid barrier of the skin by increasing the biosynthesis of intercellular lipids [16]. Shea butter contains lipids that are similar to those of the skin, thereby supporting the skin’s natural lipid layers [40]. Aqua posae filiformis is a strong emollient that has been shown to decrease pruritus and restore healthy skin microbiota [41].

The BAP Scar Care^®^ silicone gel consists of polysiloxanes or silicone polymers that provide occlusion, a smoother texture and increase spreadability. It also contains vitamin E but no hydrating ingredients. In 2013 this product was tested at our research center with 2 other widely used silicone gels: Dermatix^®^ (Meda Pharmaceuticals, Hoeilaart, Belgium) and Kelo-Cote^®^ (InTe Medical, Anderlecht, Belgium). Given that the BAP Scar Care^®^ gel proved to be the most occlusive of several silicone gels in the study by Hoeksema et al. (2013) [18], and being used in the more recent study by De Decker et al. (2022) [16], this was included as a fluid silicone reference product.

TEDRA^®^ is a water-based moisturizer that primarily contains glycerin, dimethicone (polysiloxanes) and cetearyl hexanoate (cetearyl alcohol). Cetearyl hexanoate has strong emollient properties and also provides occlusive effects, whilst glycerin is a strong humectant [36,42,43,44]. Three different compositions of the TEDRA^®^ moisturizer were tested: the commercially available composition, which is from here on called ‘TEDRA^®^’, and two compositions not commercially available: TEDRA^®^ NT1 and the TEDRA^®^ NT3. All three moisturizers contain the same ingredients, although in different proportions. Regular TEDRA^®^ has a higher proportion of silicates compared to TEDRA^®^ NT1 and NT3, while TEDRA^®^ NT3 contains a higher load of active ions compared to regular TEDRA^®^ and TEDRA NT1.

### 2.5. Statistical Analysis

Statistical analyses were performed using GraphPad Prism version 9.0.2 (San Diego, CA, USA). The normality of the data was assessed using the Shapiro–Wilk test. Data are presented as mean ± standard deviation (SD). For non-normally distributed data, pairwise comparisons between more than two groups were performed using the Friedman test. A *p* value < 0.05 was considered a priori to be statistically significant when using the Friedman test. When significant, post-hoc uncorrected Dunn’s tests for pairwise comparisons between two groups were performed. Statistical significance was set at *p* value < 0.05.

## 3. Results

### 3.1. Participants

Forty healthy volunteers were recruited for this study. None of the volunteers had known allergies of any kind to skincare products and no allergic reactions were seen throughout the study. The volunteers included 8 male and 32 female participants with a mean age of 22.68 (±1.89) years. All participants were Caucasian. Fitzpatrick skin types ranged from type I–III. Six male volunteers and none of the female volunteers’ forearms required shaving. In 4 of the 40 participants (all female), a decrease in TEWL post-stripping was documented and these volunteers were subsequently excluded from analysis. An overview of all the objective measurements that are discussed below can be found in Table 1.

### 3.2. Environmental Conditions

All measurements were performed in the same examination room throughout the study. The room had controlled and monitored temperature and humidity. Additionally, the room was isolated from the rest of the hospital and the outside world.

### 3.3. Objective Measurements

#### 3.3.1. Occlusive Properties

##### Mean TEWL

**Figure 2:**
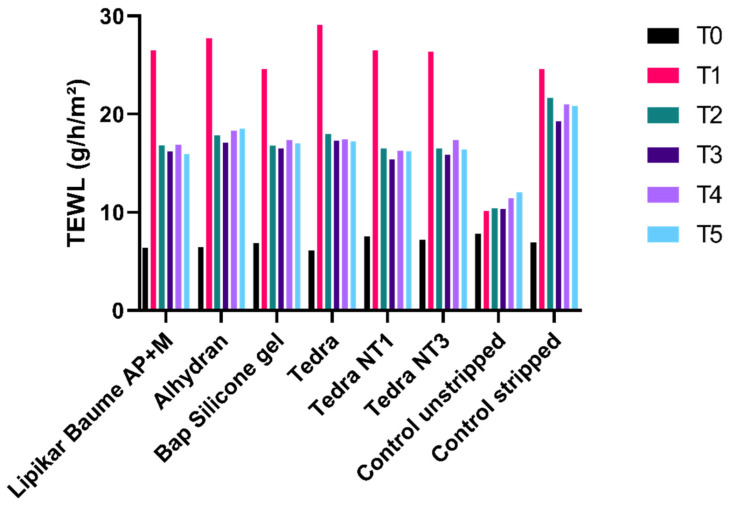
Mean TEWL values during follow-up time expressed as (g/h/m^2^).

Figure 2 illustrates the mean TEWL values measured in grams per hour per square meter (g/h/m^2^) for all moisturizers, fluid silicone gel, as well as both stripped and unstripped control sites. For the stripped controls, TEWL increased immediately after stripping (T1) and remained consistent thereafter. In contrast, the sites where moisturizers and silicone gel were applied showed a noticeable increase in TEWL right after stripping (T1). However, one hour after the application of these products, TEWL began to considerably decrease (T2). In the case of unstripped skin, TEWL values experienced a slight increase from T1 to T5, but they generally remained stable over time, with no significant differences observed between consecutive measurements (Wilcoxon; *p* > 0.05). These findings are consistent with the ambient room conditions and align with results reported in the study by De Decker et al. (2022) [16].

##### Mean Percentage Reduction (MPR)

The Mean Percentage Reduction (MPR) in TEWL was determined using the following formula. This formula considers the percentage reduction of TEWL while accounting for the baseline TEWL (T0) values and TEWL induction (T1) immediately after stripping for each individual volunteer. This calculation was performed for each hour post-application, extending up to 4 h (T2, T3, T4, and T5). It is important to note that T1 represents 0% TEWL reduction, while T0 represents 100% TEWL reduction, serving as fixed reference values (see Formula (1)).

(1) $$MPR\left(t\right)=\frac{\sum_{i=1}^{n}1-\frac{T\left(i,t\right)-T\left(i,0\right)}{T\left(i,1\right)-T\left(i,0\right)}}{n}\times 100$$

**Formula 1.** Mean Percentage Reduction (MPR)

where:
*i* = ith volunteer
*n* = total number of volunteers
*T*(*i*,*t*) = TEWL of volunteer *i* at time *t* in hours
*T*(*i*,0) = TEWL of volunteer *i* at baseline, after acclimatization
*T*(*i*,1) = TEWL of volunteer *i* post-tape stripping, application time
*MPR*(*t*) = Mean Percentage Reduction at *t* hours post-application

**Figure 3:**
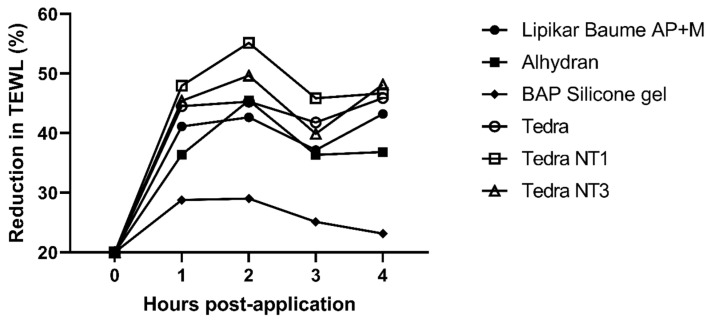
Mean Percentage Reduction (MPR) in TEWL up to 4 h after product application.

*MPR* serves as an effect size that quantifies the extent of differences in TEWL reduction values [16,18,45]. In essence, it is a statistical metric employed to gauge the impact of a treatment when compared to a control. The outcomes can be found in Table 1 and are visually depicted in Figure 3. Formula (1) adheres to a previously validated and published MPR formula [16,18,45]. The associated statistical analysis is presented in Table 2.

The fluid silicone gel (BAP silicone gel) reached its maximal MPR at two hours post-application (T3) of 29.03% (±37.00) and continued to decrease steadily thereafter. The MPR values of TEDRA^®^, TEDRA^®^ NT1 and TEDRA^®^ NT3 were (very strong or strong) significantly better than those of the MPR of the BAP silicone gel at every measurement (T2–T5), except for TEDRA^®^ NT3 at T4, which was not significantly different from the BAP scar silicone gel. TEDRA^®^, TEDRA^®^ NT1 and TEDRA^®^ NT3 were not significantly different from one another during T2-T5. However, TEDRA^®^ NT1 had the largest MPR value of all the tested products at 55.15% (±32.33). Of all the tested hydration substances in this study, BAP silicone gel had the lowest MPR of 23.15% (±57.66), measured 4 h post-application. Alhydran^®^ performed significantly better in terms of MPR than the BAP silicone gel at 2 h post-application (T3). Lipikar^®^ was not significantly different from the silicone gel, TEDRA^®^ NT3 or Alhydran^®^ at any point in time. The MPR of TEDRA^®^ NT1 was significantly better than that of Lipikar^®^ at T1 and T2.

#### 3.3.2. Skin Hydration

##### Mean Hydration Values

**Figure 4:**
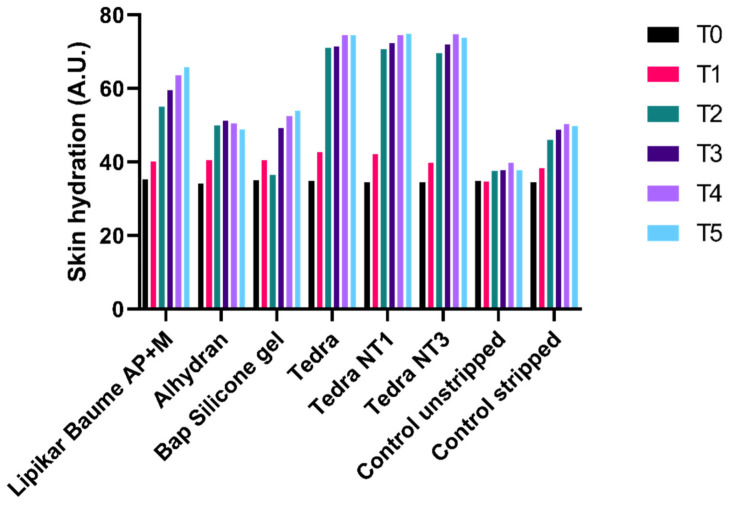
Mean hydration values as measured by the Corneometer CM825 during follow-up time. A.U. = arbitrary units.

Figure 4 displays the average hydration values, with the units measured in arbitrary units (A.U.) using the Corneometer CM 825. In comparison to the initial measurement (T0), all average hydration values exhibited an increase over time. For the unstripped control sites, mean hydration values demonstrated a gradual increase over time, likely influenced by the room’s environmental conditions. However, there were no statistically significant differences observed between consecutive measurements, as determined by the Wilcoxon paired-matches signed-ranks test (*p* > 0.05). Mean hydration values for the stripped control sites increased over time. This can be attributed to the room’s environmental conditions in conjunction with the additional hydration effect from the stripping procedure. These findings align with those reported in the study conducted by De Decker et al. (2022) [16].

##### Cumulative Absolute Added Hydration

Cumulative Absolute added hydration at time t, CAAH(t), was calculated using Formula (2):

(2) $$CAAH\left(t\right)=\frac{\sum_{i=1}^{n}1-\frac{HP\left(i,t\right)-HSC\left(i,0\right)}{HP\left(i,1\right)-HSC\left(i,0\right)}}{n}\times 100$$

**Formula 2.** Cumulative Absolute Added Hydration (CAAH)

where:
*i* = *i*-th volunteer
*n* = total number of volunteers
*HP*(*i*,*t*) = hydration after application of product *P* of volunteer *i* at time *t* in hours, which indicates that *HP*(*i*,0) = hydration after application of product *P* of volunteer *i* at application time, and that *HP*(*i*,1) = hydration after application of Product *P* of volunteer *i* 1 h after application
*HSC*(*i*,*t*) = hydration of the stripped control site of volunteer *i* at time *t* in hours, which indicates that *HSC*(*i*,0) = hydration of the stripped control site of volunteer *i* at application time
*CAAH*(*t*′) = Cumulative Added Hydration at *t* hours post-application in arbitrary units (A.U.).

**Figure 5:**
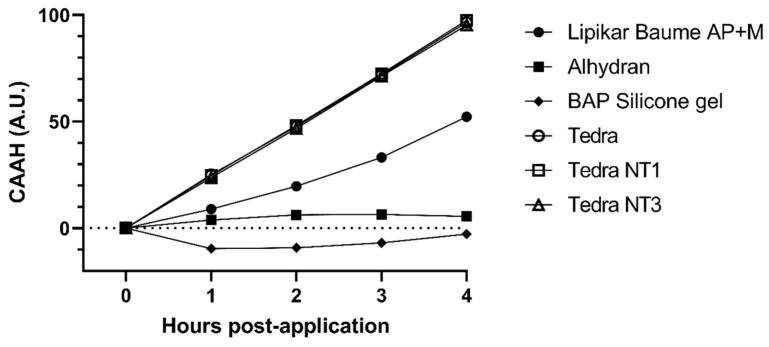
Cumulative Absolute Added Hydration (CAAH), relative to the stripped controls and expressed in arbitrary units (A.U.) as assessed by the Corneometer.

Similar to MPR, CAAH is an effect size. It represents the magnitude of the differences in hydration and is presented cumulatively. Results are shown in Table 1 and average CAAH values are illustrated in Figure 5. Statistical analysis of CAAH is shown in Table 3. Formula (2) is based on the previously validated and published CAAH formula [16,18,45].

The highest CAAH values of all the tested products were registered for TEDRA^®^, TEDRA^®^ NT1 and TEDRA^®^ NT3 with respective CAAH values of 96.52 (±53.05), 97.50 (±59.48) and 95.25 (±53.55) at T2-T5. These three moisturizers were not significantly different from one another at any point in time (*p* > 0.05). The lowest CAAH values were recorded for BAP silicone gel with a negative CAAH of −2.72 (±41.08) at T2–T5. All moisturizers showed positive CAAH values. CAAH values of Alhydran^®^ at T2–T5 were the lowest of all tested moisturizers. TEDRA^®^, TEDRA^®^ NT1 and TEDRA^®^ NT3 all had very strongly significantly higher CAAH values compared to Alhydran^®^, BAP silicone gel and Lipikar^®^ at T2–T5. With the exception of Alhydran^®^, all moisturizers had significantly higher CAAH values at T2–T5 than the silicone gel. Alhydran^®^ had significantly higher CAAH at T2 compared to BAP silicone gel. The CAAH of TEDRA^®^, TEDRA^®^ NT1, TEDRA^®^ NT3 and Lipikar^®^ increased over time, as can be seen in Figure 5.

#### 3.3.3. Microcirculatory Changes

##### Mean Perfusion Units

**Figure 6:**
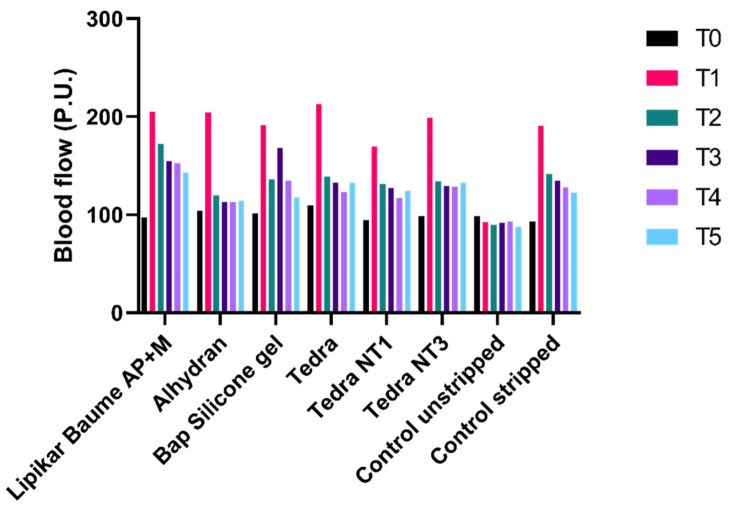
Mean blood flow values as measured by the FLPI-2 during follow-up time. P.U. = perfusion units.

Figure 6 provides a visual representation of the average perfusion values, measured in arbitrary units known as Perfusion Units (P.U.), using the FLPI-2. In comparison to the initial measurement (T0), all average perfusion values exhibited an upward trend over time. For the unstripped control sites, mean perfusion values remained relatively stable and experienced a slight decrease over time. In contrast, mean perfusion values for the stripped control sites also decreased over time, similar to the trend observed in the unstripped controls. However, the reduction in perfusion for the stripped controls was more pronounced than that seen in the unstripped controls.

##### Cumulative Absolute Difference in Blood Flow

Similar to MPR and CAAH, CADBF is an effect size that represents the magnitude of differences in perfusion and is presented in a cumulative manner [16,18,45].

Cumulative absolute difference in blood flow at time t, CADBF(t), was calculated using Formula (3):

(3) $$CADBF\left(t\right)=\frac{\sum_{i=1}^{n}1-\frac{PP\left(i,t\right)-PSC\left(i,0\right)}{PP\left(i,1\right)-PSC\left(i,0\right)}}{n}\times 100$$

**Formula 3.** Cumulative Absolute Difference in Blood Flow (CADBF)

where:
*i* = *i*-th volunteer
*n* = total number of volunteers
*PP*(*i*,*t*) = perfusion after application of product *P* of volunteer *i* at time *t* in hours, which indicates that *PP*(*i*,0) = perfusion after application of Product *P* of volunteer *i* at application time and that *PP*(*i*,1) = perfusion after application of Product *P* of volunteer *i* 1 h after application
*PSC*(*i*,*t*) = perfusion of the stripped control site of volunteer *i* at time *t* in hours, which indicates that *PSC*(*i*,0) = perfusion of the stripped control site of volunteer *i* at application time
*CADBF*(*t*′) = Cumulative Absolute Difference in Blood Flow at *t* hours post-application in arbitrary perfusion units [P.U.].

**Figure 7:**
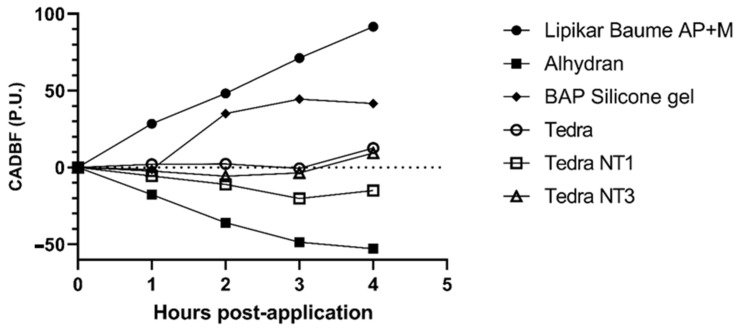
Cumulative absolute differences in blood flow (CADBF) in perfusion units (P.U.) of the different moisturizers relative to the stripped control sites as assessed by Laser Speckle Contrast Imaging (FLPI-2).

The results are shown in Table 1 and the average CADBF values are illustrated in Figure 7. The results of the statistical analysis of the CADBF values are listed in Table 4.

**Figure 8:**
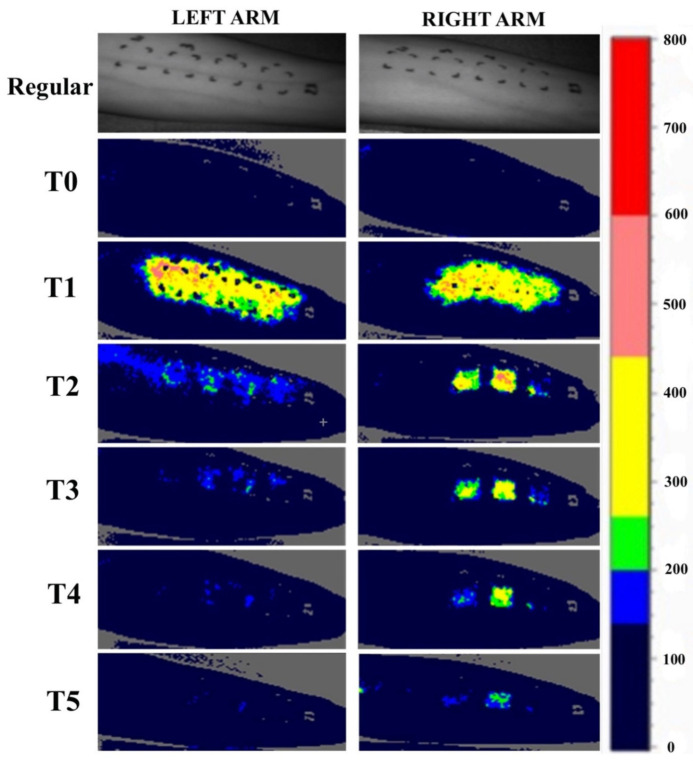
Differences in blood flow over time in one individual as assessed with LSCI over time (T0–T5). Left arm (**upper left image**), areas from left to right on the image: Alhydran^®^, TEDRA^®^ NT3, TEDRA^®^ and TEDRA^®^ NT1. Right arm (**upper right image**), areas from left to right on the image: unstripped control, stripped control, Lipikar^®^ and Bap^®^ Silicone gel.

According to the CADBF, Lipikar^®^ and BAP silicone gel resulted in a relatively strong increase in blood flow over time (T2-T5) with the highest value of 91.64 (±124.15). Alhydran^®^ and TEDRA^®^ NT1 both showed a decrease in blood flow over time. Alhydran^®^ showed the strongest decrease in blood flow with a CADBF of −52.83 (±136.17). TEDRA^®^ and TEDRA^®^ NT3 were almost stable over time, only resulting in a limited increase in blood flow at T2–T5 with, respectively, 12.56 (±118.87) and 9.36 (±174.65). TEDRA^®^, TEDRA^®^ NT1 and TEDRA^®^ NT3 were not statistically different from one another in terms of changes in blood flow at any point in time. However, TEDRA^®^ NT1 showed the greatest decrease in CADBF. The CADBF of the silicone gel was not statistically different from TEDRA^®^, TEDRA^®^ NT1 and TEDRA^®^ NT3. Alhydran^®^ showed significantly reduced CADBF compared to TEDRA^®^, TEDRA^®^ NT3, Lipikar^®^ and BAP silicone gel. Figure 8 represents the changes in blood flow over time at every time point (T0–T5), as evaluated through LSCI, for a study participant. The observed fluctuations in blood flow over time demonstrate a strong correlation with the overall results on CADBF, as depicted in Figure 7.

## 4. Discussion

The topic of scar management is highly complex and clinicians can approach it in various ways. One common piece of advice often given to patients with scars is to moisturize the scar regularly. Despite the widespread acceptance and frequent recommendation of this approach, little evidence exists regarding the effectiveness of moisturizers on scars. Clinicians are unsure about the effects of the products they recommend on scars, as there is limited research available on the topic [1,46]. This limited research available to date has primarily relied on subjective assessments of scars. To address this knowledge gap, the aim of this study was to investigate the effects of moisturizers on scars in an objective way. Specifically, the study aimed to measure the effect of moisturizers on three key parameters that are commonly altered in scars: TEWL, hydration and microcirculation of the skin.

Previous studies investigated the effect of moisturising products on TEWL and hydration and have shown that some of the products tested were able to produce the favourable effects of a decrease in TEWL and an increase in hydration [16,18]. The primary objective of the current study was to investigate whether the tested moisturizers can induce changes in microcirculation and whether there is a correlation with the previously established effects of these moisturizing products on scars, by incorporating the measurement of microcirculation.

Rodrigues et al. (2004) [47] occluded the microcirculation in the forearm with a pressure cuff for three minutes and then released the cuff, measuring TEWL before, during and after occlusion, demonstrating a slight increase in TEWL from baseline during occlusion and then a decrease with reactive hyperemia and stabilization. This suggests a negative correlation between microcirculation and TEWL. However, caution should be exercised when extrapolating these findings to moisturizers, as the study involved complete occlusion of microcirculation, which differs from partial local and topical influence. The underlying mechanism for this relationship was not explained. Conversely, Agner et al. (2000) [48], while examining a patch test model, found that an irritant (SLS) decreased hydration and increased TEWL and blood flow. However as the study was run over two days, the increase in TEWL and blood flow was attributed to an inflammatory reaction [48].

The effects of Alhydran^®^, BAP Silicone gel and Lipikar^®^ have been previously measured [16]; however, it was noted that Alhydran^®^ did not achieve as much of an increase in hydration as previously observed [16]. The reason for this discrepancy remains unknown as all environmental and test conditions remained constant and similar to the previous study. BAP Silicone gel demonstrated similar results to the previous study [16]. Lipikar showed increased occlusive properties compared to the previous study. Alhydran^®^ reduced TEWL, which was accompanied by a steady decrease in blood flow over the four hours, exhibiting an opposite effect compared to the other products. However, for active scars this outcome is considered preferable.

Lipikar^®^ was able to decrease TEWL over the study period but only over the first two time points was this significantly different to TEDRA^®^ NT1. Lipikar^®^ was able to increase hydration more effectively than Alhydran^®^, which became significant at the last two time points and was significantly greater than the BAP Silicone gel at all time points. The absence of hydration with the silicone gel is attributable to the absence of moisturizing and humectant components. Despite Lipikar^®^ demonstrating moderate performance in terms of TEWL and hydration, it caused a significantly greater increase in blood flow compared to all other products tested. Clinically, it has been observed that patients using this moisturizer report a tingling sensation. This may be a result of the presence of niacinamide, a derivative of nicotinamide, which stimulates an inflammatory reaction [49] (see Appendix A). Niacinamide has been observed in other studies to elicit mild burning, pruritis and erythema, but these side effects are only experienced by a small percentage of patients [50,51]. However, an increase in blood flow is not the preferred outcome for active scars, as erythema reduction is preferred.

Three formulations of TEDRA^®^ were tested to determine their relative efficacy, considering variations in ion and silicate concentrations. The TEDRA^®^ products all performed similarly, with indistinguishable hydration values among them. Additionally, they were also more effective in reducing TEWL than the other tested products. When comparing the pharmaceutical formulations of the different products, TEDRA^®^ has a good ratio of humectants, emollients and occlusives (see Appendix A). In contrast, Alhydran incorporates occlusives, humectants, and emollients, with the emollients providing additional occlusion. Notably, the significant presence of Aloe Vera in Alhydran, a potent moisturizer, is expected to contribute to enhanced hydration, as evidenced in previous studies. While the precise mechanism behind Alhydran’s reduction of blood flow remains unclear, it represents a notable advantage. This is most likely attributable to the cooling effects supplied by the moisturizing components in combination with the water-based formulation. Additionally, the pronounced anti-itching effect of Alhydran is likely linked to this reduction in blood flow. Based on its formulation, Lipikar^®^ has many occlusive and humectant ingredients but relatively lacks emollients. With TEDRA^®^, there was minimal change in blood flow over the four-hour period. This particular product was selected for study due to its affordability and some clinical observations of itch reduction. It was initially anticipated that a decrease in blood flow would be observed, but contrary to expectations, this was not the case.

BAP silicone gel exhibited the lowest effectiveness in normalizing and reducing TEWL, with its effect gradually diminishing over the four-hour period. However, it is noted that a slightly lower TEWL induction was measured at the BAP silicone gel site. This is resolved with the MPR formula as it accounts for the baseline (T0) and the TEWL induction post-striping (T1) with T1 considered as 0% TEWL reduction. BAP silicone gel was the only product to reduce hydration in the tape-stripped skin, with the skin almost approaching baseline at the end of the four-hour period. These results are similar to what was observed when this product was previously tested [16]. It must be noted that this product is formulated with ingredients that do not include emollients or humectants. Additionally, it does not contain water or any other moisturizing component in contrast to the other products, therefore explaining the negative CAAH values in contrast to the moisturizers. Instead of water or moisturizing components, it contains polysiloxanes with limited occlusive properties and vitamin E for anti-oxidation, resulting in the expected low occlusive effects. Studies have shown that polysiloxanes have significant anti-scar activity when used in combination with moisturizing and humectant components, as is the case with Lipikar and TEDRA, but not in the absence of these ingredients, such as with silicone gel, thereby explaining the results of the study [34]. The study demonstrated inferior occlusive effects of the silicone gel compared to the tested moisturizers. Furthermore, due to the absence of emollients and humectants, no true barrier repair is expected. The effect of the silicone gel on blood flow became apparent after two hours, when an increase in blood flow was observed. The combination of results indicates that the BAP Silicone Gel was the least effective among the tested products in influencing the investigated parameters. This conclusion is consistent with the findings of our previous study [16].

This current study provided evidence that moisturizers influence the blood flow. However, the effects on blood flow varied across the products tested. The effect on blood flow did not correlate to the extent of changes in TEWL and hydration. It cannot yet be concluded whether the observed effects on blood flow, either increased or decreased, have a positive effect on scar formation or other clinical outcomes when combined with interventions such as pressure garments. A decrease in blood flow is proposed to be a preferable change, as it may potentially decrease itching and reduce scar hypertrophy. In the case of active scars, the preferred effect is to not only normalize TEWL and increase hydration but also to decrease microcirculation, as scars tend to have higher blood perfusion compared to normal skin and mature scars.

TEDRA^®^ and Alhydran^®^ were found to have a cooling effect upon application, but this is merely an observation based on the subjective perception of the test subjects. Conducting studies that measure subtle variations in skin temperature can provide insights into potential differences among moisturizers. Moreover, additional research is necessary to investigate the long-term effects of extended moisturizer application on the restoration of the barrier function of scarred skin through objective measurements. Such studies are important and would help shed light on the role of these intricate formulations in scar management.

The impact of moisturizers on scar blood flow in patients undergoing pressure therapy, which effectively reduces microcirculation in the skin, remains uncertain. However, the combined use of moisturizers and pressure therapy is crucial and supported by their ability to provide occlusive and hydrative properties, contributing to a well-balanced and adequately hydrated state of the skin. Unfortunately, it is important to recognize that the majority of scars do not receive pressure therapy due to various factors, such as patient non-compliance, reimbursement limitations, and unfavourable climate conditions. In such cases, the significant reduction in blood flow demonstrated by specific moisturizers in this study is expected to offer substantial benefits. These findings underscore the potential value of moisturizers in scar treatment, especially when pressure therapy is not feasible or not being implemented.

While the evidence available supports the use of moisturizers in scar management, the effect of moisturizers on the microcirculation remains unclear. Further research is required to elucidate the relationship between moisturizers, microcirculation, and scar formation. It is possible that moisturizer products contain ingredients that have a more substantial impact on stratum corneum repair and scar modulation than what is measured by their effects on blood flow, hydration, and TEWL.

### Limitations of the Study

In this study, the impact of moisturizers on parameters such as TEWL, hydration, and skin microcirculation was measured. However, an important parameter, repair of the barrier function of the stratum corneum, cannot be determined in this model.

While the standard deviations in Table 1 may lead to concerns regarding measurement reliability, they largely stem from inherent individual variability within the model. To mitigate this, standardized formulas were employed and the priority was intra-individual consistency. Despite potential influences from external factors, rigorous control measures were used. Notably, the study’s robust statistical power, derived from a significant number of intra-individual tests, and the significance of the results collectively reinforce the overall validity and reliability of our findings.

## 5. Conclusions

This study demonstrated that moisturizers influence the microcirculation of the skin, with some moisturizers causing the desired decrease in microcirculation for scar treatment, while others induce a presumed less desirable increase in blood flow. However, the specific added value for scar outcomes remains unclear in contrast to the clinical effects of effective hydration and occlusion, which are clearly evident. Further research is necessary to investigate the clinical impact of reducing microcirculation on scars by the application of moisturizers, both when applied in combination with and without the use of pressure therapy. Additionally, potential adverse clinical effects of increased microcirculation caused by certain moisturizers on hypertrophic scar formation should also be investigated.

## Figures and Tables

**Table 1:** Mean and standard deviation (SD) of mean percentual reduction (MPR) in trans epidermal water loss (TEWL), cumulative absolute added hydration (CAAH) and cumulative absolute difference in blood flow (CADBF).

Data Overview												
	TEDRA^®^		TEDRA^®^ NT1		TEDRA^®^ NT3		Alhydran^®^		BAP Silicone Gel		Lipikar^®^ Baume	
	Mean	SD	Mean	SD	Mean	SD	Mean	SD	Mean	SD	Mean	SD
**MPR in TEWL % (*n* = 36)**												
T2	44.53	19.13	47.96	31.65	45.42	35.47	36.38	33.99	28.78	44.86	41.10	26.39
T3	45.27	23.67	55.15	32.33	49.66	38.26	45.48	19.67	29.03	37.00	42.66	36.02
T4	41.77	31.44	45.86	29.15	39.89	40.73	36.36	26.43	25.13	43.81	37.15	43.28
T5	45.87	23.79	46.68	42.42	48.15	46.43	36.82	30.87	23.15	57.66	43.23	31.63
**CAAH A.U. (*n* = 36)**												
T2	25.05	13.06	24.66	14.91	23.60	14.33	3.86	12.65	−9.57	16.64	8.91	16.57
T2–T3	47.62	25.22	48.16	29.68	46.79	28.19	6.28	21.98	−9.13	26.96	19.67	32.49
T2–T4	71.82	38.37	72.46	45.37	71.31	41.10	6.45	32.33	−6.86	35.03	33.10	46.98
T2–T5	96.52	53.05	97.50	59.48	95.25	53.55	5.51	41.82	−2.72	41.08	52.20	59.31
**CADBF P.U. (*n* = 36)**												
T2	2.00	36.11	−5.54	37.79	−2.36	43.16	−17.63	42.59	−1.31	50.84	28.40	51.28
T2–T3	2.33	60.59	−10.98	70.02	−5.68	85.42	−35.99	75.02	35.14	256.38	48.16	79.86
T2–T4	−0.59	100.65	−20.21	121.21	−3.51	142.93	−48.59	117.04	44.41	261.18	71.22	103.41
T2–T5	12.56	118.87	−14.95	141.70	9.36	174.65	−52.83	136.17	41.70	269.07	91.64	124.15

**Table 2:** Mean Percentual Reduction (MPR) in Trans Epidermal Water Loss (TEWL).

Data Overview					
Reduction of TEWL (*n* = 36)			Friedman Test *p*-Value ^a, b^		
T2			**0.0176**		
T3			**0.0074**		
T4			**0.0444**		
T5			**0.0441**		
	**Post hoc test *p*-value ^d^**				
	**Reduction of TEWL T2 ^c^ (*n* = 36)**	**Reduction of TEWL T3 ^c^ (*n* = 36)**		**Reduction of TEWL T4 ^c^ (*n* = 36)**	**Reduction of TEWL T5 ^c^ (*n* = 36)**
TEDRA^®^ vs. Alhydran^®^	0.6041	0.8969		0.2705	0.0801
TEDRA^®^ vs. Lipikar^®^	0.5169	0.7954		0.4758	0.5596
TEDRA^®^ vs. BAP	**0.0381**	**0.0445**		**0.0095**	**0.0165**
TEDRA^®^ vs. TEDRA^®^ NT1	0.1360	0.0695		0.6041	0.7954
TEDRA^®^ vs. TEDRA^®^ NT3	0.8458	0.4758		0.4367	0.7459
Alhydran^®^ vs. Lipikar^®^	0.8969	0.6973		0.6973	0.2433
Alhydran^®^ vs. BAP	0.1198	**0.0324**		0.1360	0.5169
Alhdyran vs. TEDRA^®^ NT1	**0.0445**	0.0919		0.1051	0.1360
Alhydran^®^ vs. TEDRA^®^ NT3	0.4758	0.5596		0.7459	**0.0381**
Lipikar^®^ vs. BAP	0.1539	0.0801		0.0601	0.0695
Lipikar^®^ vs. TEDRA^®^ NT1	**0.0324**	**0.0381**		0.2181	0.7459
Lipikar^®^ vs. TEDRA^®^ NT3	0.3994	0.3309		0.9483	0.3642
BAP vs. TEDRA^®^ NT1	**0.0004**	**0.0001**		**0.0019**	**0.0324**
BAP vs. TEDRA^®^ NT3	**0.0233**	**0.0065**		0.0695	**0.0065**
TEDRA^®^ NT1 vs. TEDRA^®^ NT3	0.1948	0.2705		0.1948	0.5596

^a^ = Friedman test compares the 6 products every hour after product application; ^b^ = Statistically significant if *p* < 0.05; ^c^ = A significant Friedman test was followed by the correct post-hoc testing; ^d^ = Uncorrected Dunn test was used.

**Table 3:** Cumulative absolute added hydration (CAAH).

Data Overview					
CAAH (*n* = 36)		Friedman Test *p*-Value ^a, b^			
**T2**		**<0.0001**			
T2–T3		**<0.0001**			
T2–T4		**<0.0001**			
T2–T5		**<0.0001**			
	**Post hoc test *p*-value ^d^**				
	**CAAH T2 ^c^ (*n* = 36)**		**CAAH T2–T3 ^c^ (*n* = 36)**	**CAAH T2–T4 ^c^ (*n* = 36)**	**CAAH T2–T5 ^c^ (*n* = 36)**
TEDRA^®^ vs. Alhydran^®^	**<0.0001**		**<0.0001**	**<0.0001**	**<0.0001**
TEDRA^®^ vs. Lipikar^®^	**<0.0001**		**<0.0001**	**<0.0001**	**<0.0001**
TEDRA^®^ vs. BAP	**<0.0001**		**<0.0001**	**<0.0001**	**<0.0001**
TEDRA^®^ vs. TEDRA^®^ NT1	0.6735		0.7459	0.6041	0.6973
TEDRA^®^ vs. TEDRA^®^ NT3	0.6269		0.8969	>0.9999	0.8969
Alhydran^®^ vs. Lipikar^®^	0.1539		0.0518	**0.0233**	**0.0165**
Alhydran^®^ vs. BAP	**0.0381**		0.1198	0.2181	0.4758
Alhdyran vs. TEDRA^®^ NT1	**<0.0001**		**<0.0001**	**<0.0001**	**<0.0001**
Alhydran^®^ vs. TEDRA^®^ NT3	**<0.0001**		**<0.0001**	**<0.0001**	**<0.0001**
Lipikar^®^ vs. BAP	**0.0005**		**0.0005**	**0.0005**	**0.0019**
Lipikar^®^ vs. TEDRA^®^ NT1	**<0.0001**		**<0.0001**	**<0.0001**	**<0.0001**
Lipikar^®^ vs. TEDRA^®^ NT3	**<0.0001**		**<0.0001**	**<0.0001**	**<0.0001**
BAP vs. TEDRA^®^ NT1	**<0.0001**		**<0,0001**	**<0.0001**	**<0.0001**
BAP vs. TEDRA^®^ NT3	**<0.0001**		**<0.0001**	**<0.0001**	**<0.0001**
TEDRA^®^ NT1 vs. TEDRA^®^ NT3	0.9483		0.6500	0.6041	0.6041

^a^ = Friedman test compares the 6 products every hour after product application; ^b^ = Statistically significant if *p* < 0.05; ^c^ = A significant Friedman test was followed by the correct post-hoc testing; ^d^ = Uncorrected Dunn test was used.

**Table 4:** Cumulative absolute difference in blood flow (CADBF).

Data Overview					
CADBF (*n* = 36)		Friedman Test *p*-Value ^a, b^			
**T2**		**<0.0001**			
T2–T3		**<0.0001**			
T2–T4		**<0.0001**			
T2–T5		**<0.0001**			
	**Post hoc test *p*-value ^d^**				
	**CADBF T2 ^c^ (*n* = 36)**		**CADBF T2–T3 ^c^ (*n* = 36)**	**CADBF T2–T4 ^c^ (*n* = 36)**	**CADBF T2–T5 ^c^ (*n* = 36)**
TEDRA^®^ vs. Alhydran^®^	**0.0053**		**0.0023**	0.0518	**0.0043**
TEDRA^®^ vs. Lipikar^®^	**0.0087**		**0.0095**	**0.0023**	**0.0053**
TEDRA^®^ vs. BAP	0.1123		0.3642	0.6973	0.5596
TEDRA^®^ vs. TEDRA^®^ NT1	0.2181		0.1734	0.1198	0.0518
TEDRA^®^ vs. TEDRA^®^ NT3	0.6041		0.6973	0.9483	0.8969
Alhydran^®^ vs. Lipikar^®^	**<0.0001**		**<0.0001**	**<0.0001**	**<0.0001**
Alhydran^®^ vs. BAP	0.2305		**0.0324**	**0.0196**	**0.0233**
Alhdyran vs. TEDRA^®^ NT1	0.1198		0.0919	0.6973	0.3642
Alhydran^®^ vs. TEDRA^®^ NT3	**0.0233**		**0.0079**	**0.0445**	**0.0065**
Lipikar^®^ vs. BAP	**<0.0001**		**0.0005**	**0.0079**	**0.0007**
Lipikar^®^ vs. TEDRA^®^ NT1	**0.0001**		**<0.0001**	**<0.0001**	**<0.0001**
Lipikar^®^ vs. TEDRA^®^ NT3	**0.0017**		**0.0029**	**0.0029**	**0.0035**
BAP vs. TEDRA^®^ NT1	0.7215		0.6500	0.0518	0.1734
BAP vs. TEDRA^®^ NT3	0.2848		0.6041	0.7459	0.6500
TEDRA^®^ NT1 vs. TEDRA^®^ NT3	0.4758		0.3309	0.1051	0.0695

^a^ = Friedman test compares the 6 products every hour after product application; ^b^ = Statistically significant if *p* < 0.05; ^c^ = A significant Friedman test was followed by the correct post-hoc testing; ^d^ = Uncorrected Dunn test was used.

## Data Availability

All data are presented in the main manuscript.

## Appendix A

**Table A1:** Product Ingredients and Their Function. * Unknown or Nil Evidence to Suggest Indication of Ingredient.

Alhydran^®^	Function	Lipikar^®^	Function	TEDRA^®^	Function	BAP Scar Care^®^ Silicone Gel	Function
**Aloe Barbadensis Leaf Juice (Aloe Vera gel from Aruba)**	Moisturizer	**Aqua/Water**	Solvent	**Aqua**	Solvent	**Poly-siloxanes**	Occlusive, enhance spread ability
**Water**	Solvent	**Butyrospermum Parkii Butter/Shea Butter**	Moisturizer	**Glycerin**	Humectant	**Vitamin E**	Antioxidant
**Ceteraryl Alcohol**	Emollient	**Glycerin**	Humectant	**Ceteraryl ethyl-hexanoate**	Emollient		
**Caprylic/Capric Triglyceride**	Emollient	**Dimethicone**	Occlusive	**C14–22 Alcohols**	Emollient		
**Decyl Oleate**	Emollient	**Niacinamide**	Vitamin B3	**C10–18 Triglycerides**	Emollient		
**Glyceryl Stearate**	Emulsifier	**Paraffin Liquid/Mineral Oil**	Occlusive	**Sodium Polyacrylate**	Thickening agent		
**Cetearyl Isonoanoate**	Emollient	**Cetearyl Alcohol**	Emollient	**Dimethicone**	Occlusive		
**Sorbitan Stearate**	Emulsifier	**Rapeseed Oil (Brassica Campestris Oleifera Oil)**	Moisturizer	**Phenoxyethanol**	Preservative		
**Ceteareth-20**	Emulsifier	**Ammonium Polyacryldimethyltauramide/Ammonium Polyacryloyldimethyl Taurate**	Thickening agent	**Tocopheryl Acetate—Vitamin E**	Antioxidant		
**Propylene Glycol**	Humectant	**PEG-100 Stearate**	Emulsifier	**C12–20 Alkyl Glucoside**	Emulsifier		
**Phenoxyethanol**	Preservative	**Glyceryl Stearate**	Emulsifier	**Caprylyl Glycol**	Preservative		
**Ceteareth-12**	Emulsifier	**Peg-20 Methyl Glucose Sesquistearate**	Emulsifier	**Chorphenesin**	Preservative		
**Cetyl Palmitate**	Emollient	**Cera Microcristallina/Microcrystalline Wax**	Binding Agent	**Dimethiconol**	Texture/Spread ability		
**Caprylyl Glycol**	Humectant	**Paraffin**	Occlusive	**Hydrochloric Acid**	pH adjustment (Stability)		
**Chlorphenesin**	Preservative	**Sorbitan Tristearate**	Emulsifier	**Trideceth-6**	Emulsifier		
**Tocopheryl Acetate—Vitamin E**	Antioxidant	**Dimethiconol**	Texture/Spread ability	**Manganese Chloride Tetrahydrate**	*		
**Ascorbic Acid—Vitamin C**	Antioxidant	**Mannose**	Moisturizer	**Potassium Chloride**	Fluid Balance		
**Jojoba Oil (Simmondsia Chinensis Seed Oil)**	Emollient	**Disodium EDTA**	Chelating agent (adds stability)	**Sodium Metasilicate Pentahydrate**	*		
**Disodium EDTA**	Chelating agent (adds stability)	**Capryloyl Glycine**	Antimicrobial	**Magnesium Chloride Hexahydrate**	*		
		**Vitreoscilla Ferment**	Moisturizer	**Lithium Chloride**	*		
		**Xanthan Gum**	Thickening Agent				
		**Pentaerythrityl Tetra-Di-T-Butyl Hydroxy-hydrocinnamate**	Antioxidant (stability)				
		**Sodium Benzoate**	Preservative				
